# The Positive Effect of Negative Stimuli: Exposure to Negative Emotional Stimuli Improves Mood in Individuals with Major Depressive Disorder

**DOI:** 10.3390/jcm14176189

**Published:** 2025-09-02

**Authors:** Sapir Miron, Eldad Keha, Eyal Kalanthroff

**Affiliations:** 1 Department of Psychology, The Hebrew University of Jerusalem, Jerusalem 91905, Israel; eldad.keha@mail.huji.ac.il; 2 Department of Psychology, Achva Academic College, Arugot 7980400, Israel; 3 Department of Psychiatry, Columbia University Irving Medical Center, New York, NY 10032, USA

**Keywords:** depression, emotion, cognition, mood, information processing

## Abstract

**Background**: Cognitive biases in information processing, particularly attentional and memory biases, play a crucial role in the development and maintenance of Major Depressive Disorder (MDD). These biases lead individuals with MDD to preferentially attend to and remember negative information, thereby maintaining a depressed mood. A recently proposed attentional resources model suggests that exposure to negative stimuli leads to deeper cognitive processing of subsequent information, regardless of its content. Based on this model, the current study investigated a novel paradigm that manipulated exposure to negative emotional stimuli and examined its effect on information processing and mood improvement. **Method**: Thirty-eight unmedicated participants with MDD and no comorbid disorders, and 37 healthy controls, completed three blocks of an emotional recall task, which involved watching a short emotional video followed by a recall task of neutral or positive valence stories. Mood changes were assessed throughout the task. **Results**: Results revealed that both the MDD and HC groups reported improved mood after exposure to a negative emotional video followed by a positive story. **Conclusions**: These results have important clinical implications. The paradigm may be applied in a broader sense as an active tool that may help to improve mood in depression treatment.

## 1. Introduction

Major Depressive Disorder (MDD) is a highly prevalent and debilitating mental health condition characterized by persistent feelings of sadness, loss of interest in activities, and various cognitive and physical symptoms [1,2,3,4]. Despite a range of interpersonal, psychotherapeutic, and pharmacological interventions, treatment outcomes for MDD are moderate, and many individuals experience relapses [5,6,7] (This emphasizes the importance of understanding the mechanisms underlying the maintenance of MDD as well as identifying new tools to augment existing treatments [8]. Cognitive models of depression share the premise that attentional biases play a significant role in the development and maintenance of MDD [9,10,11,12]. These attentional biases are characterized by selectively attending to negative information, having difficulty disengaging from it, and avoiding allocating attention to positive information [13,14,15,16,17]. Attentional bias is also associated with memory bias, which leads to increased recall of negative information, thereby significantly contributing to the maintenance of a depressed mood [18].

Studies have suggested that interventions targeting attentional biases, known as ‘cognitive bias modification,’ are effective in altering these biases and, consequently, alleviating symptoms of MDD [19,20]. Specifically, Attention Bias Modification, which uses repeated practice aimed at enhancing the processing of positive stimuli and shifting attention away from negative stimuli, has shown effectiveness in altering negative attentional bias and reducing MDD symptoms [20,21]. Interestingly, Woolridge et al. (2021) [22] found that Attention Bias Modification training also increased positive memories in individuals with MDD. Targeting the memory bias directly, researchers found that different Memory Bias Modification tools are effective in altering memory bias in MDD [23] and reducing negative mood in those individuals [24].

**Figure 1:**
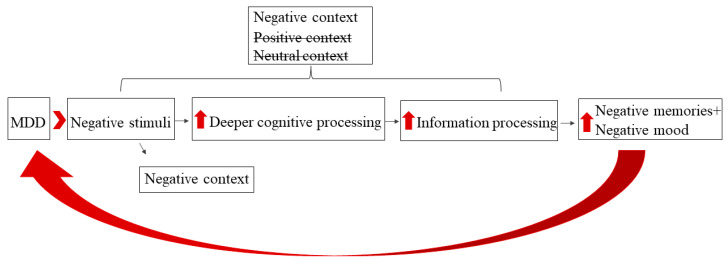
The cognitive resources model. Due to attentional bias, individuals with MDD attend to negative cues in the environment. In turn, these cues lead to deeper cognitive processing, which leads to greater information processing, especially of negative information that exists in the context of the original negative stimuli. Such deeper processing leads to increased negative memories and negative mood [25]. Upward arrow indicates increasing levels.

Despite the promising evidence that cognitive bias modification interventions can reduce both attention and memory biases and alleviate symptoms of MDD, additional tools are required to enhance patients’ ability to process and remember positive information. Recently, a new ‘cognitive resources model’ for MDD has been proposed (Figure 1; [25,26]. According to this model, when an individual with MDD encounters a negative cue, it triggers the recruitment of cognitive resources, leading to deeper cognitive processing of subsequent information. This notion is based on Craik and Lockhart’s (1972) [27] classic model, which posits that highly familiar and meaningful stimuli trigger higher/deeper levels of processing. Thus, negative information serves as a meaningful stimulus and triggers an ‘attentional window’ of deeper cognitive processing, especially for individuals with MDD. For example, Miron et al. (2020) [26] found that negative cues presented to individuals with high levels of depression led to improved performance in the illusory conjunction task, which necessitates high levels of information processing. Furthermore, deeper cognitive processing during these ‘attentional windows’ leads to better recall of this information. Miron and Kalanthroff (2024) [28] found that participants with MDD exhibited enhanced memory recall of neutral and even positive information when it was preceded (primed) by a negative-valence video compared to when it was preceded by a neutral or a positive video. Importantly, this model does not contradict the attentional bias model as it agrees with the notion that individuals with MDD are drawn to negative cues and suggests that during these ‘attentional windows,’ individuals with MDD will be drawn to mood-congruent negative cues. However, this model goes beyond the cognitive bias model by suggesting that in circumstances where the ‘attentional window’ is open due to an encounter with a negative cue, and no other subsequent negative information is present, all available information will be processed deeper and remembered better, even if this information is neutral or positive (see Figure 1). Interestingly, while this effect has been observed in all individuals, it is more pronounced in those with MDD [29]. 

While it has been shown that negative cues can open ‘attentional windows’ for deeper cognitive processing and memorization of subsequent information, especially in individuals with MDD, the clinical implications of this effect have not yet been tested. Therefore, the goal of the current study was to take the first step toward utilizing the cognitive resources scientific model (Figure 1) to improve the mood of individuals diagnosed with MDD. Such a proof of concept could lead to the future development of novel interventions and enhance cognitive treatments for MDD. For that aim, we administered a novel paradigm in which unmedicated patients with MDD and no comorbid disorders, as well as healthy controls (HCs), were asked to memorize and recall a positive or a neutral story after being exposed to either negative, positive, or neutral emotional prime stimuli. Mood changes were assessed using a Visual Analogue Scale (VAS) throughout the procedure. Based on the theoretical model, we hypothesized that exposure to negative emotional prime stimuli would improve the processing and memorization of subsequent information regardless of its content. Furthermore, we hypothesized that in the condition where participants are exposed to negative emotional prime stimuli and the subsequent information is positive, their mood would be elevated due to enhanced processing of the positive information. While we expected to see this effect in all participants, we anticipated it would be significantly stronger in the MDD group. Additionally, as previous studies have indicated that positive cues might lead to reduced processing in MDD (the opposite effect of negative cues), we hypothesized that positive information following positive prime stimuli would be processed and remembered less effectively and would have less impact on mood.

## 2. Methods

### 2.1. Participants

Thirty-eight unmedicated adults diagnosed with MDD and no comorbid disorders, and 37 HCs participated in the study in return for a small monetary compensation (~USD 11). The study was approved by the institutional ethics board (HUJI-2021-03171). Informed consent was obtained from all subjects involved in the study prior to their participation in the study. All participants were between the ages of 19 and 36, were either currently enrolled in or had completed their undergraduate studies, had no current psychiatric disorders (other than MDD in the MDD group), and were naive naïve to the purposes of the experiment. Initial screening was conducted using an online survey with four items taken from the Beck Depression Inventory-II (BDI-II; [30]), which was completed by approximately 4850 university students as part of a larger screening survey. An invitation email was sent to individuals who had a total score of 12 (maximum score) and to individuals who had a total score of 4 or lower. Upon arriving at the lab, all participants completed a full BDI-II questionnaire and underwent a Structured Clinical Interview based on the Diagnostic Interview for Anxiety, Mood, and OCD and Related Neuropsychiatric Disorders (DIAMOND; [31]) to substantiate diagnosis. The interview was conducted by a licensed clinical psychologist, supervised by a licensed senior clinical psychologist (Ph.D.) experienced in the assessment and treatment of MDD. Out of all the individuals who replied to the invitation, only participants with BDI-II scores > 15, who met DSM-5 criteria for MDD, had no other current co-morbid psychopathology, and were not on any psychiatric or neurological medication for at least 6 months, were included in the MDD group. Participants with BDI-II scores < 6 who had no current or history of MDD and had no other current or past co-morbid psychopathology were included in the HC group. The BDI-II total score ranges from 0 to 63. This laborious diagnostic process allowed us to have a well-diagnosed sample with MDD with minimal potential artifacts and a HC group with similar demographics: the final sample included 38 patients with MDD (30 females, 8 males, mean age = 23.71, SD = 3.37, mean BDI-II = 24.92, range = 15–41), and 37 HC participants (23 females, 14 males, mean age = 23.70, SD = 1.61, mean BDI-II = 2.86, range = 0–6). The mean BDI-II score for the MDD group falls within the moderate depression range.

A power analysis using G * Power 3.1 [32], based on the effect size reported in a previous study that compared MDD and HC participants in a memory task (η^2^ = 36; [33]), indicated that the current sample allowed for examination of group differences in recall performance at a power > 95% to test small–medium effect size with a Type 1 error (α < 0.05).

### 2.2. Procedure

**Figure 2:**
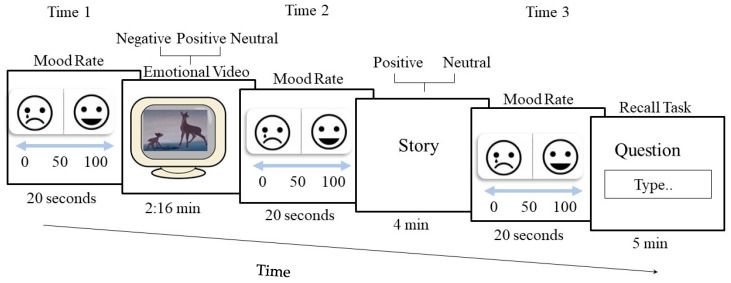
Example of a block of the emotional recall task. Participants were required to watch a short video (negative, neutral, or positive valence), read a story (positive or neutral), and finally answer 10 memory questions about the story. Participants were asked to rate their current mood on a Visual Analogue Scale at three time points.

Data collection and stimulus presentation were controlled by an HP EliteDisply 800G3TWR computer with an Intel i7-8700 4.20GHz processor (HP Inc., Palo Alto, CA, USA; Intel Corp., Santa Clara, CA, USA). The stimuli were presented on an HP EliteDisplay E240 LED 23.8” monitor (HP Inc., Palo Alto, CA, USA; Intel Corp., Santa Clara, CA, USA). Participants were tested individually in a quiet laboratory testing room. After signing an informed consent form, participants underwent a clinical interview, completed the BDI-II, and then completed the *emotional recall task* (see Figure 2). At the end of the study, participants were paid, thanked, and debriefed.

### 2.3. The Emotional Recall Task (Figure 2)

First, participants were required to watch a two-minute and sixteen-second emotional video (negative, neutral, or positive) selected from a standardized, validated video archive to maximize the emotional impact and “remember as many details as possible” [34,35]. For a happy video, we used “Bean Camera” (from the ‘Mr. Bean goes to town’ episode; [36]), which was rated as inducing high levels of amusement (M = 5.78). For a sad video, we selected a scene from “Bambi” [37]), which was rated as inducing high levels of sadness (M= 5.35). For a neutral video, we selected a scene that represented fish in the aquarium that was chosen as inducing little report of emotions based on criteria recommended by Rottenberg et al. (2007) [38]. Next, participants were asked to read a short story (neutral or positive). Due to ethical and scientific considerations, we did not include a negative (video)–negative (story) condition. The goal of the current study was to demonstrate deeper processing of positive information and not of negative information, which is already a very well-known phenomenon in MDD. In addition, following this study’s hypotheses (and the existing literature on attention bias and cognitive processing in MDD), a negative–negative condition might have had negative consequences on our participants with MDD (adapted from Bishop et al., 2004 [39]). There were three core stories (“park,” “beach,” and “going home”), each available in two different valence versions (positive or neutral). Each story consisted of three parts: the first part was constant across all versions, while the other two parts varied by version. The changes in valence were achieved by altering a few key words in the last two parts of each story, ensuring minimal potential artifacts. All stories were between 328 and 354 words in length, with versions of the same story differing by no more than 8 words. Each participant completed three blocks, with each block containing one video of a different emotional valence (negative, neutral, and positive), such that each participant was exposed to all three valence videos. The order of blocks was randomized across participants. Overall, there were six possible blocks comprising different combinations of video valence and story valence. To reduce burden, each participant completed only one block with each emotional video (a total of three blocks), with the story valence randomly selected for each block. The full texts of the stories used in each condition are available in the Supplementary Materials. To confirm task engagement, participants answered 10 memory questions about the story at the end of each block, using a textbox. Mood was reported at the beginning of the task (Time 1), after watching the video (Time 2), and after reading the story (Time 3) using a Visual Analogue Scale (VAS) using the computer mouse, with ratings ranging from sad to happy. Participants had to drag it to continue. Participants were given a 30-second break between the blocks (see Figure 2). The task duration was comparable across participants, as all components were time-limited and standardized per block.

## 3. Results

The original data presented in the study are openly available in OSF, at https://osf.io/9735v/ (accessed on OSF: 04 August 2024). Demographic characteristics of the sample by group are described in the Section 2.1. There were no significant differences in age between groups, *t*(223) = 0.022, *p* = 0.982. There were significant differences in gender (χ^2^(1) = 7.664, *p* = 0.006, Cramer’s V = 0.006).

As expected, the memory test was very easy and participants answered correctly on average 7.3 (SD = 1.6) memory questions (out of 10 questions), indicating proper task engagement. To identify manipulation, we carried out a mixed-model analysis of variance (ANOVA) on VAS mood data at Time 2 (after watching the video) minus Time 1 (at the beginning of the task) with video valence (negative vs. neutral vs. positive) as a within-subject factor and group (HC vs. MDD) as a between-subject factor. The results revealed a significant main effect of video valence (*F*(2, 146) = 65.83, *p* < 0.001, $\eta_{p}^{2}$ = 0.474), no significant main effect of group (*F*(1, 73) = 0.32, *p* = 0.572), and no significant interaction (*F*(2, 146) = 0.43, *p* = 0.652). Planned comparisons revealed higher rating (more positive mood at Time 2 compared to Time 1) for the positive video condition compared to the neutral video condition (*t*(74) = 2.56, *p* = 0.013, Cohen’s *d* = 0.30), and lower ratings (more negative mood at Time 2 compared to Time 1) for the negative video condition compared to the neutral video condition (*t*(74) = 8.45, *p* < 0.001, Cohen’s *d* = 0.98). Taken together, these results indicate that the video manipulation influenced mood as expected according to its condition, with no differences between the groups.

**Figure 3:**
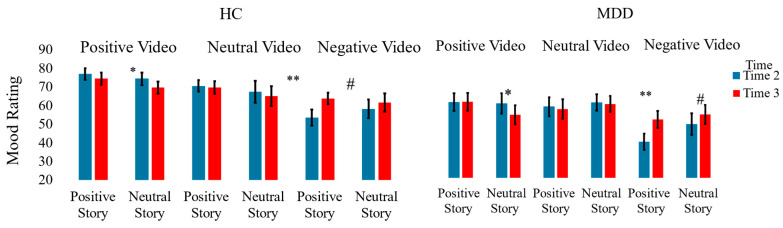
Mean mood ratings with error bars representing +/− 1 standard error of the mean. Time 2 represents the mood rating after watching the video, while Time 3 represents the mood rating after reading the story. Higher ratings indicate better mood, while lower ratings indicate worse mood. * *p* < 0.05, ** *p* < 0.001, **#** marginally significant. The significant effects did not differ between the groups.

To test our main hypothesis, we employed a linear mixed-effects model (LMM) analysis using the *lme4* package 1.1-35.4 in R studio 4.4.1 to predict VAS mood data based on group, valence, story valence (neutral vs. positive), and time point (Time 2 (after watching the video) vs. Time 3 (after reading the story)). We first fitted a null model to assess baseline mood variation across participants and time points, accounting for within-subject random effects *(time|sub)*. Random effects indicate that we allowed intercepts and slopes to vary across subjects, capturing individual differences in VAS mood responses. Subsequently, we developed a series of increasingly complex linear mixed-effects models to examine how VAS mood was influenced by the different factors (for the complete model summary table, see Supplementary Materials). Model 1 included fixed effects for group, valence, story valence, and time point, with random effects accounting for variability in VAS mood trajectories over time within-subject specified as *(time|sub)* (see Figure 3). Building upon this, Model 2 introduced interactions between valence, story valence, and time point as fixed effects, maintaining the same random effect structure (see Figure 3). Model 3 further extended this by integrating group into the interaction terms (valence X story valence X time point X group), allowing for differential effects of video valence and story valence over time across the MDD and HC groups (see Figure 3). For a detailed formulation of each model, please refer to the Supplementary Materials, where the complete model specifications and their formulas are provided.

Model 1, which included only the main effects, was significant (χ^2^(3) = 135.719, *p* < 0.001). The predicted estimate of mood for the HC group was significantly larger (more positive) than the predicted estimate of mood in the MDD group (estimate = 13.82, *p* < 0.001). In addition, the positive video predicted higher scores compared to the neutral video (estimate = 2.83, *p* = 0.011), and the negative video predicted lower scores compared to the neutral video (estimate = 9.76, *p* < 0.001). Finally, neither story valence (estimate = 0.64, *p* = 0.504) nor time point (estimate = 1.12, *p* = 0.212) predicted mood.

Model 2, which included the three-way interaction, was significant (χ^2^(7) = 45.516, *p* < 0.001). To further explore the three-way interaction, we tested whether the difference between VAS mood data at Time 2 and Time 3 changed as a function of the interaction between video valence and story valence. To calculate these contrasts, we used the *emmeans* package (Lenth, 2018, [40]). The results showed that, following a negative video, there was a non-significant increase in VAS mood rating in the neutral story condition (*t*(364) = 1.876, *p* = 0.061, Cohen’s d = 0.098) and a significant increase in the positive story condition (*t*(364) = 5.365, *p* < 0.001, Cohen’s d = 0.281). The increase in VAS in the positive story condition was significantly greater than the increase in VAS in the neutral story condition (*t*(364) = 2.294, *p* = 0.022, Cohen’s d = 0.120. Following the positive video, there was a decrease in VAS mood rating following a neutral story (*t*(364) = 2.418, *p* = 0.016, Cohen’s d = 0.127), but not following the positive story (*t*(364) = 0.556, *p* = 0.578). There was no significant difference between the effects of the neutral and positive stories following a positive video (*t*(364) = 1.386, *p* = 0.166). Finally, following the neutral video, there was no change in VAS mood rating, both in the neutral (*t*(364) = 0.742, *p* = 0.458) and the positive (*t*(364) = 0.462, *p* = 0.644) story conditions.

Finally, since Model 3, which tested the four-way interaction, was not significant (χ^2^(11) = 14.155, *p* = 0.224), we did not explore this interaction further and deduced that the three-way interaction tested in Model 2 did not significantly differ between the HC and MDD groups.

## 4. Discussion

The current study aimed to examine the clinical implications of a new model of attentional resources. To this end, we employed an innovative paradigm that directed participants’ attention toward neutral or positive information after priming their cognitive system with negative, neutral, or positive stimuli. Specifically, participants were asked to watch a video with negative, neutral, or positive valence before being asked to read a story with positive or neutral valence. Our findings support the notion that exposure to negative emotional stimuli creates temporal ‘attentional windows’ that facilitate cognitive processing of subsequent information—participants showed the most significant mood improvement after viewing a negative emotional video, followed by a positive story. Remarkably, this mood improvement surpassed that observed in the positive (video) positive (story) condition. Contrary to our hypothesis, this effect was comparable in both the HC and MDD groups.

Previous studies have shown that emotional stimuli trigger enhanced attention and processing of mood-congruent information [11,41]. In real-life contexts, this attentional bias often results in the predominant processing of negative information, as negative information often presents itself in the context of the original negative trigger. However, in our controlled study, presenting positive information after negative stimuli led to subsequent mood improvement. These results cannot be accounted for by the classic attentional bias model, as they indicate that attentional resources were manipulated, not just the scope of these attentional resources. Furthermore, our results suggest that while negative stimuli effectively open an ‘attentional window,’ positive stimuli achieve the opposite effect and lead to reduced attentional processing resources. These results align with previous research indicating better memory recall following exposure to negative cues [25,26] and extend these findings by demonstrating the utility of this model in affecting mood. The latter is consistent with existing literature indicating that individuals with MDD exhibit an attenuated attentional bias for positive information [16,17,42,43,44] ( as well as reduced recall of positive information [45]. For example, studies have shown that positive autobiographical memories in individuals with MDD are often less vivid and emotionally intense [46,47], and their recall is generally reduced [48]. The most important finding of the current study is that these fluctuations in attentional resources, caused by the introduction of negative or positive cues, can be manipulated. In other words, while positive information per se is mostly ignored, it can be deeply processed if the system is ‘tricked’ into deeper processing by the presence of a negative prime.

Recognizing cognitive mechanisms that perpetuate negative mood by facilitating the processing and recall of negative information, while ignoring positive content, is vital. However, our results provide empirical support not only for the theoretical model suggesting that attentional resources fluctuate in response to exposure to valenced cues, but also for the notion that deeper cognitive processing of positive information is a key mechanism for mood improvement in MDD. Our results identify a cognitive mechanism that may enhance the processing of such positive information. Hence, these results may have potential clinical implications for developing targeted interventions to improve depression treatment outcomes. The manipulation presented here could potentially serve as a novel therapeutic tool integrated at the beginning of treatment sessions to enhance the processing of positive information by first triggering negative emotions. This approach may help generate representations of positive information in memory, thus breaking the cycle of negative feelings. Additionally, it could address memory biases in MDD and aid interventions targeting autobiographical memory biases. Although our results provide initial support for this clinical implication, future studies should explore these potential clinical methods and ideas before they can be used in the clinic.

Contrary to our expectations, the effect of the negative prime on attentional resources and mood improvement through positive information did not significantly differ between participants with MDD and HCs. While we anticipated this pattern in HCs based on prior studies [25] and observed a marginally larger effect in the MDD group, this difference did not reach statistical significance. This unexpected finding, though not entirely consistent with our model, aligns with previous research demonstrating mood improvement in non-depressed individuals when recalling positive autobiographical memories after negative mood induction [49]. Furthermore, another study by Rusting and DeHart (2000) [50] found that recalling positive memories after experiencing negative affective states can elevate mood in non-depressed individuals. These results suggest that negative cues may have a similar effect on both HCs and individuals with MDD, indicating that our proposed model might not be exclusive to MDD but rather a more general mechanism of the emotional effect on attentional resources. However, based on extensive literature, we maintain that this effect is likely more robust and significant in MDD, although this was not clearly evident in the current sample. Further research is needed to determine the model’s specificity to MDD.

The well-diagnosed sample of unmedicated MDD patients with no comorbid disorders, as well as the model-driven hypothesis and method, represent significant strengths of the current study. However, there are notable limitations that should be acknowledged. First, the lack of long-term mood measurement precludes proper assessment of the long-lasting effects of the manipulation. Second, the omission of a negative story condition limits our ability to investigate the full spectrum of our theoretical model. However, the negative story condition was excluded for both theoretical and ethical reasons; specifically, the goal was to enhance positive processing rather than to demonstrate the well-documented effect of excessive negative information processing in MDD. Third, our MDD sample consisted of unmedicated individuals without comorbid disorders. While this strict scientific approach increases internal validity by minimizing potential confounding effects, it reduces the generalizability of the findings, as most clinical MDD patients present with comorbidities such as anxiety. Finally, the current study aimed to test the effect of information processing manipulation on mood. Accordingly, the memory test was intentionally simple and served only as a manipulation check. It did not allow for a direct assessment of the manipulation’s effect on information processing—an effect that has been demonstrated in previous research [28]. We also acknowledge that the simplicity of the task may have resulted in a ceiling effect. Such an effect could have influenced mood, for example, by inducing a sense of success following high performance, particularly following the negative video. This may be especially relevant for participants in the MDD group. Accordingly, this possibility should be considered when interpreting the results. While our conclusions focus specifically on individuals diagnosed with MDD, future research may explore whether the mechanism of leveraging attentional windows to enhance mood can be generalized to non-clinical or subclinical populations.

In conclusion, our findings offer a novel perspective on cognitive processing in MDD, highlighting the potential of attentional windows as a mechanism for enhancing the impact of positive information. The overall pattern of our suggested model shows that negative emotional cues may create a cognitive opportunity for deeper engagement with subsequent positive content, and thereby improve mood. This mechanism has the potential to improve the efficacy of treatments for depression.

## Data Availability

The data presented in this study are openly available in Open Science Framework at https://osf.io/9735v/. accessed on OSF: 04 August 2024).

## Supplementary Materials

The following supporting information can be downloaded at: https://www.mdpi.com/article/10.3390/jcm14176189/s1, Table S1: Summary of linear mixed models predicting mood, including fixed and random effects, across four model iterations. Table S2: Model formulae used for linear mixed models in R. Table S3: Accuracy rates for the recall memory task by story valence, video valence, and group. Table S4: Correlation analysis between memory performance and mood rates. Text S1: Story scripts used in the emotional recall task.
