# Supplementary material for: The Positive Effect of Negative Stimuli: Exposure to Negative Emotional Stimuli Improves Mood in Individuals with Major Depressive Disorder
Source: J Clin Med. 2025 Sep 2;14(17):6189. doi: 10.3390/jcm14176189 (PMC12429692; doi:10.3390/jcm14176189)
Supplement: Supplementary file 1 [file jcm-14-06189-s001.zip › jcm-3807501-supplementary.pdf]

Supplementary Material

Models Summary Table

Table S1. Linear Mixed Models Predicting Mood

	**mood**		**mood**		**mood**		**mood**	
Predictors	*Estimates*	*p*	*Estimates*	*p*	*Estimates*	*p*	*Estimates*	*p*
(Intercept)	59.93	**<0.001**	68.47	**<0.001**	67.70	**<0.001**	65.94	**<0.001**
group [MDD]			−13.82	**<0.001**	–13.61	**0.001**	–10.27	**0.043**
emotional [negative]			–9.76	**<0.001**	–9.03	**<0.001**	–6.15	**0.039**
emotional [positive]			2.83	**0.011**	7.36	**0.007**	9.73	**0.010**
story [positive]			0.64	.504	4.62	0.088	5.95	0.116
time [3]			1.12	.212	–1.50	0.459	–2.32	0.428
time [3] × emotional [negative]					5.56	0.061	5.77	0.158
time [3] × emotional [positive]					–3.73	0.209	–2.41	0.566
time [3] × story [positive]					0.50	0.866	1.48	0.724
emotional [negative] × story [positive]					–10.25	**0.001**	–14.26	**0.001**
emotional [positive] × story [positive]					–7.33	0.105	–6.10	0.332
(time [3] × emotional [negative]) × story [positive]					6.29	0.134	5.30	0.372
(time [3] × emotional [positive]) × story [positive]					3.60	0.390	0.71	0.904
group [MDD] × time [3]							1.55	0.700

group [MDD] × emotional [negative]							–5.88	0.172
group [MDD] × emotional [positive]							–4.57	0.399
group [MDD] × story [positive]							–2.44	0.652
(group [MDD] × time [3]) × emotional [negative]							–0.14	0.982
(group [MDD] × time [3]) × emotional [positive]							–2.60	0.660
(group [MDD] × time [3]) × story [positive]							–1.90	0.748
(group [MDD] × emotional [negative]) × story [positive]							7.65	0.218
(group [MDD] × emotional [positive]) × story [positive]							–2.37	0.794
(group [MDD] × time [3] × emotional [negative]) × story [positive]							1.55	0.854
(group [MDD] × time [3] × emotional [positive]) × story [positive]							5.61	0.502
**Random Effects**								
σ^2	125.12		90.86		81.81		81.09	
τ_{00}	303.13 $_{sub}$		273.09 $_{sub}$		278.29 $_{sub}$		281.01 $_{sub}$	
τ_{11}	0.23 $_{sub.time3}$							
ρ_{01}	1.00 $_{sub}$							
ICC			0.75		0.77		0.78	
N	75 $_{sub}$		75 $_{sub}$		75 $_{sub}$		75 $_{sub}$	

Observations	450	450	450	450
Marginal R^2 / Conditional R^2	0.000 / NA	0.175 / 0.794	0.193 / 0.817	0.197 / 0.820

Table S2. Models Formulae.

Null model

```
null_model <– lmer(mood ~ 1 + (time | sub), data = data)
```

Model 1

```
model1 <– lmer(mood ~ group + emotional + story + time + (1 | sub), data = data)
```

Model 2

```
model2 <– lmer(mood ~ group + time * emotional * story + (1 | sub), data = data)
```

Model 3

```
model23 <– lmer(mood ~ group + time * emotional * story * group + (1 | sub), data = data)
```

Text S1: Story Scripts Used in the Emotional Recall Task.

The following are the full texts of the three stories used in the experimental blocks. These were selected and adapted from validated materials used in prior research (e.g., Bishop et al., 2004) and presented following each emotional video.

THE PARK STORY

Section 1. Text

One Saturday, the sun was shining and the day was warm. There were a lot of children in the playground. A few parents were there as well. Some of the children were waiting to use the slide. Others were playing on the roundabout. Jane had just come off the swings. She wanted to find something else to do. She decided to get an ice cream. Some of Jane's friends stayed to play in the playground. Others went to the river where they could paddle. Some left on their own. The rest left with older brothers or sisters. Jane thought that there was a shop that sold ice cream nearby. She walked to the shop. When she got there, she asked the shopkeeper if he sold ice cream. The

shopkeeper said "yes" and Jane bought one. It was the sort that she liked best. She left the shop, and ate her ice cream on a bench outside. It was nice and she sang to herself as she set off back to the playground.

Section 2. Text: positive version

There were two ways that Jane could walk back. There was the way that she had got to the shop. But there was also a quicker way. Jane decided to take the quicker way. Her parents had walked this way with her before. It was quiet and quite sunny. Jane felt happy. She started to sing more loudly and skipped along up the path. She walked past a house with a fence round its back garden. She heard a noise and turned around. A boy stepped out through a door in the fence. He was a friend from school. He had something in his hand. It was a baby squirrel. Jane looked at it, feeling amazed. "Put out your arms," said the boy. He moved forward, holding the baby squirrel. Jane agreed and put her arms out. The boy gave the baby squirrel to her. Jane laughed out loud and felt excited. "Come with me," said the boy, touching Jane's hand. "Walk with me to the park." The park was close by. Jane walked beside the boy, feeling very happy. Just as they reached the park, Jane saw her Uncle. He lived on the other side of the park, and must have been for a walk. Jane called out to him. The boy looked surprised. "Who's that?" he said. Jane's Uncle joined them. He watched while Jane put the baby squirrel down. Then he shook the boy's hand, and smiled at him. "You must come back with us," he said. "Let's find Jane's parents and tell them what you've been doing. Rescuing a baby squirrel!" The boy looked proud. Jane's Uncle put a hand on his shoulder and the three of them left the park.

Section 2. Text: neutral version

There were two ways that Jane could walk back. There was the way that she had got to the shop. But there was also a quicker way. Jane decided to take the quicker way. Her parents had walked this way with her before. It was quiet and quite cloudy. Jane felt tired. She started to sing more loudly and walked on up the path. She walked past a house with a fence round its back garden. She heard a noise and turned around. A boy stepped out through a door in the fence. He was someone from school. He had something in his hand. It was a small conker. Jane looked at it, feeling puzzled. "Put out your hand," said the boy. He moved forward, holding the small conker. Jane shrugged and put her hand out. The boy gave the small conker to her. Jane took it and felt surprised. "Come with me," said the boy, tapping Jane's arm. "Walk with me to the park." The park was close by. Jane walked beside the boy, feeling very tired. Just as they reached the park, Jane saw her Uncle. He lived on the other side of the park, and must have been for a walk. Jane called out to him. The boy looked interested. "Who's that?" he said. Jane's Uncle joined them. He watched while Jane put the small conker down. Then he shook the boy's hand, and nodded at him. "You must come back with us," he said. "Let's find Jane's parents and tell them what you've been doing. Finding conkers!" The boy looked tired. Jane's Uncle put a hand on his shoulder and the three of them left the park.

THE BEACH STORY

Section 1. Text

One day in July, Ben was at the beach. His parents and some of his friends were there too. The weather was good. There were a lot of people sunbathing. Some children were building sandcastles. Ben was paddling in the sea. He came out of the water, dried his feet and put on his shoes.

Then he decided to explore the beach a bit. He looked around. Two women were sitting by the beach wall. Some children were climbing rocks. And a group of teenagers were drinking coke in front of the beach café. Ben thought that there might be some rock pools on the other side of the rocks. So he walked over to the rocks and started to climb up. He found one small pool in a hole. Some seawater had been left there when the tide was in. He looked for shells in the pool and found several that he liked. Then he began to climb further up. He still wanted to get to the top and down the other side.

Section 2. Text: positive version

The rocks were quite high. Ben found some cracks that he could put his feet in and went higher and higher. After a few minutes, he stopped for a rest. He had climbed rocks with his parents before and it was still sunny, so he felt happy. Just then, he heard a shout. It was a boy slightly older than him. “Come on,” the boy said. “I’ll help you climb.” They climbed on together and reached the top. Ben looked down the other side. Suddenly he peered forward. He felt excited. He hurried down the rocks, and reached the ground. He laughed, and was amazed. There were coins all over the ground. They were quite big. He felt very happy. He wished his parents were there but didn’t want to climb back to find them. He looked at the coins, picked some up and thought about what he should do. Just then the boy appeared. He had been climbing down slowly. He looked surprised when he saw the coins and told Ben there was a quicker way back to the main beach. He offered to show it to him. Ben got up and they walked back together. Ben’s parents had been waiting for him. They waved when they saw him and came over. Ben told his mum about the coins. His mum smiled and said, “You could be rich!” Then she turned to the boy and thanked him for helping Ben climb. The boy looked proud and asked if he could stay with them for a bit.

Section 2. Text: neutral version

The rocks were quite high. Ben found some cracks that he could put his feet in and went higher and higher. After a few minutes, he stopped for a rest. He had climbed rocks with his parents before but it had got cloudy and he felt unsure. Just then, he heard a shout. It was a boy slightly older than him. “Come on,” the boy said. “I’ll help you climb.” They climbed on together and reached the top. Ben looked down the other side. Suddenly he peered forward. He climbed down the rocks and reached the ground. He looked and saw a pile of plastic bottles and some food wrappers. There was a broken bucket and some stones. The boy appeared again and told Ben there was a quicker way back to the main beach. Ben followed him, and they walked together. When Ben got back, his parents were waiting. His mum asked him where he’d been. Ben pointed back to the rocks. “Just climbing,” he said. Then the boy waved and left.

THE GOING HOME STORY

Section 1. Text

One day in autumn, the time was half past three. At Hampton School the bell went. The last lesson ended and the children left their desks. Sally had just had a Maths lesson. She had been given some homework to do. She wanted to get a good mark for it. Some of the children stayed on after school to play football. They went to the changing rooms. Others went to the computer room to do their homework. The rest left to go home. Some left by bike, others walked. A few were picked up by their parents. Sally thought that her homework would get a good mark if it were neat. So she

went to the computer room. She did her homework on one of the computers. It looked very neat and she felt pleased. She got up and said goodbye to the children who were still there. Then she left to walk home.

Section 2. Text: positive version

Sally's parents let her walk home. She did this each day. She had to go up one road, around a bend, and along another road to get to her house. As she set off, Sally looked ahead. The trees were waving in the wind, and it was quite sunny. She felt happy, and started to skip. A bit later, she thought about what mark she'd get for her homework. This took her mind off the walk home. Suddenly Sally saw a car. It was being driven by Clive Zachs. Clive Zachs was a TV star. He lived nearby and knew Sally's parents. He was on his way home. He had had a busy day, and was thinking about how it had gone. He got closer to where Sally was standing. Sally was amazed. She waved at the car and tried to get Clive Zachs' attention. Clive Zachs put on his brakes. Sally smiled. The car stopped by Sally. She waited in front of it, feeling excited. Clive Zachs got out. He came over to Sally, to see how she was. He was surprised to see Sally and offered to take her home. Sally felt very happy. She said, "Yes" and got into the car. Clive Zachs drove Sally home. Sally's parents came out to meet them. Sally's mum hugged Clive Zachs. She thanked him for bringing Sally home. Clive Zachs looked proud. "I'd better go now," he said and left.

Section 2. Text: neutral version

Sally's parents let her walk home. She did this each day. She had to go up one road, around a bend, and along another road to get to her house. As she set off, Sally looked ahead. The trees were waving in the wind, and it was quite cloudy. She felt tired, and kept on walking. A bit later, she thought about what mark she'd get for her homework. This took her mind off the walk home. Suddenly Sally saw a car. It was being driven by Clive Zachs. Clive Zachs was Sally's brother's teacher. He lived nearby and knew Sally's parents. He was on his way home. He had had a busy day, and was thinking about how it had gone. He got closer to where Sally was standing. Sally was surprised. She waved at the car and tried to get Clive Zachs' attention. Clive Zachs put on his brakes. Sally watched. The car stopped by Sally. She waited in front of it, feeling bored with walking. Clive Zachs got out. He came over to Sally, to see how she was. He was interested in how things were going and offered to take Sally home. Sally felt very tired. She said, "Yes" and got into the car. Clive Zachs drove Sally home. Sally's parents came out to meet them. Sally's mum greeted Clive Zachs. She thanked him for bringing Sally home. Clive Zachs looked tired. "I'd better go now," he said and left.

Table S3. Accuracy Rates for the Recall Memory Task by Story Valence, Video Valence and Group.

Group	Video Valence	Story Valence	Mean Rating	SE Rating
HC	Neutral Video	Neutral Story	0.721	0.044
HC	Neutral Video	Positive Story	0.828	0.033
HC	Negative Video	Neutral Story	0.695	0.041
HC	Negative Video	Positive Story	0.776	0.028

HC	Positive Video	Neutral Story	0.733	0.037
HC	Positive Video	Positive Story	0.763	0.027
MDD	Neutral Video	Neutral Story	0.714	0.045
MDD	Neutral Video	Positive Story	0.700	0.043
MDD	Negative Video	Neutral Story	0.720	0.035
MDD	Negative Video	Positive Story	0.696	0.040
MDD	Positive Video	Neutral Story	0.741	0.042
MDD	Positive Video	Positive Story	0.743	0.035

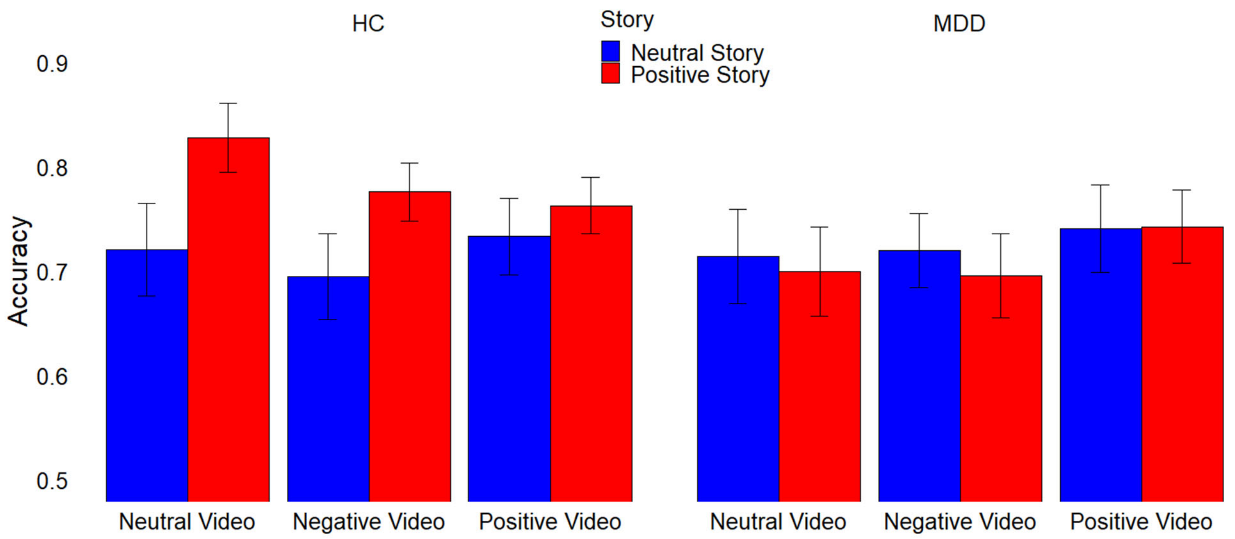

Table S4 - Correlation Analysis between memory performance and mood rates

In addition to our main analysis, we also tested all possible correlations between memory performance and mood rates. We tested whether each of the mood rates in each of the three time-points (separately for the positive and neutral stories blocks) was correlated with the recalling accuracy of the negative or positive stories. As can be seen in the table below, none of these correlations were statistically significant. In addition, we tested the correlation between the difference between time 3 and time 2 mood rates measures (separately for the positive and neutral stories blocks) and recalling accuracy of the negative or positive stories. Once again, none of the correlations were significant.

Mood Measure	Memory Measure	Correlation (r)	p-value
Neutral Story Time 1	Neutral Story Accuracy	0.111	0.343
Neutral Story Time 1	Positive Story Accuracy	−0.056	0.635
Neutral Story Time 2	Neutral Story Accuracy	0.189	0.105
Neutral Story Time 2	Positive Story Accuracy	−0.022	0.852
Neutral Story Time 3	Neutral Story Accuracy	0.18	0.121
Neutral Story Time 3	Positive Story Accuracy	0.021	0.855
Positive Story Time 3	Positive Story Accuracy	−0.152	0.193
Positive Story Time3	Neutral Story Accuracy	0.044	0.709
Positive Stroy Time 1	Neutral Story Accuracy	0.056	0.631
Positive Stroy Time 1	Positive Story Accuracy	−0.12	0.304
Positive Stroy Time 2	Neutral Story Accuracy	−0.005	0.969
Positive Stroy Time 2	Positive Story Accuracy	−0.069	0.558
Neutral Story: Time 3 - Time 2	Neutral Story Accuracy	−0.052	0.659
Neutral Story: Time 3 - Time 2	Positive Story Accuracy	0.135	0.248
Positive Story: Time 3 - Time 2	Neutral Story Accuracy	0.125	0.286
Positive Story: Time 3 - Time 2	Positive Story Accuracy	−0.214	0.065
